# USP7 Attenuates Endoplasmic Reticulum Stress and NF-*κ*B Signaling to Modulate Chondrocyte Proliferation, Apoptosis, and Inflammatory Response under Inflammation

**DOI:** 10.1155/2022/1835900

**Published:** 2022-04-06

**Authors:** Xiaofei Dong, Chang Yang, Yao Luo, Wei Dong, Xiaoxiao Xu, Yanru Wu, Jiawei Wang

**Affiliations:** ^1^The State Key Laboratory Breeding Base of Basic Science of Stomatology (Hubei-MOST) & Key Laboratory of Oral Biomedicine Ministry of Education, School & Hospital of Stomatology, Wuhan University, Wuhan, Hubei 430079, China; ^2^Nanjing Stomatological Hospital, Medical School of Nanjing University, Nanjing, Jiangsu 210000, China

## Abstract

The purpose of this research was to observe the functions and mechanisms of ubiquitin-specific peptidase 7 (USP7) on chondrocytes under tumor necrosis factor alpha- (TNF-*α*-) induced inflammation. Knee osteoarthritis (OA) models of mice were constructed by anterior cruciate ligament transection. The knee joint of mice was observed by histological staining, and the expression of USP7 was measured by immunohistochemistry staining. After knocking down or inhibiting USP7, chondrocyte proliferation was measured by histological staining and the CCK-8 assay; apoptosis was measured by western blot, flow cytometry, Caspase-3 activity, and TUNEL staining; and inflammatory response was measured by qRT-PCR and ELISA. The 4-phenylbutyric acid (4-PBA), siRNA of CHOP (si-CHOP), and QNZ were used to verify the signaling pathways. It was found that USP7 was reduced in the knee joint cartilage of OA mice. The knockdown of USP7 or its inhibitor decreased chondrocyte proliferation and accelerated apoptosis and inflammatory response under inflammation. The USP7 inhibitor exacerbated cartilage destruction in mice with OA. The knockdown of USP7 or its inhibitor activated the BiP-eIF2*α*-ATF4-CHOP signaling of endoplasmic reticulum stress (ERS) and NF-*κ*B/p65 signaling. 4-PBA, si-CHOP, and QNZ partly reversed chondrocyte proliferation, apoptosis, and inflammatory response caused by USP7 knockdown. In conclusion, through inhibiting the BiP-eIF2*α*-ATF4-CHOP signaling of ERS and NF-*κ*B/p65 signaling, USP7 promotes chondrocyte proliferation and suppresses the apoptosis and inflammatory response under TNF-*α*-induced inflammation.

## 1. Introduction

Deubiquitinases play essential roles in various diseases by modulating the posttranslational modification of related proteins. There are six families of deubiquitinases, and the largest one is the ubiquitin-specific peptidase (USP) family. Among the nearly 60 members of the USP family, ubiquitin-specific peptidase 7 (USP7) is researched extensively [1]. Increasing researches have confirmed that USP7 regulates multifaceted key protein deubiquitination to affect cell differentiation, tissue development, and disease occurrence [2, 3].

As a chronic degenerative disease of articular cartilage, osteoarthritis (OA) is commonly found with pain and dysfunction of joints and is increasing in incidence worldwide [4]. Although OA is often considered as a noninflammatory arthropathy, increased secretion of inflammatory cytokines has been observed in many patients and animal models [5]. Increasing evidence has found that proinflammatory cytokines secreted by the synovium and chondrocytes link closely to the cartilage destruction, and inflammatory pathways are vital in the progression of OA [6, 7].

Chondrocytes are the target of the inflammatory stimulation since they are the principal cells in the articular cartilage. The inflammation from OA induces chondrocyte apoptosis, which accelerates the progression of OA [8, 9]. It was found that hypoxia-inducible factor-1 alpha (HIF-1*α*) stabilization inhibited chondrocyte apoptosis and alleviated cartilage degradation in a surgical OA model [10]. Previous research has indicated that USP7 inhibits HIF-1*α* ubiquitinatioin [11]. Moreover, the activation of inflammasome plays an important role during the progression of OA, and USP7 was reported to regulate the inflammasome activation in macrophages [12, 13]. Therefore, it is reasonable to suggest that USP7 may regulate chondrocytes under inflammation, but how USP7 exerts its effect is still unknown. A previous study has found that USP7 modulated nuclear factor-kappa B (NF-*κ*B) signaling [14]. The aberrant activation of NF-*κ*B signaling was often observed in OA, and the inhibition of NF-*κ*B signaling suppressed chondrocyte apoptosis and delayed the progression of OA [15–17]. Collectively, USP7 may modulate NF-*κ*B signaling and be an important adjustor of chondrocytes under inflammation.

On the other hand, inflammation-induced endoplasmic reticulum stress (ERS) acts a key role in OA and is positively related to the cartilage degradation [18]. PERK is one of three ERS sensors involved in the BiP-PERK-eIF2*α*-ATF4-CHOP signaling of ERS [19]. Under pathological conditions, BiP binds to misfolded proteins and dissociates with PERK, which further promotes eIF2*α* phosphorylation and the subsequent high expression of ATF4 and CHOP [20]. Curcumin attenuated OA via inhibiting PERK-eIF2*α*-CHOP signaling in rats [21]. Cartilage-specific autophagy promoted PERK-ATF4-CHOP signaling to hinder the growth plate development in vivo and increase chondrocyte apoptosis and decrease chondrocyte proliferation in vitro [22]. In addition, Sirtuin-1 (SIRT1) has been reported to promote the chondrogenesis of growth plate by inhibiting the PERK-eIF2*α*-CHOP pathway, and USP7 was found to stabilize SIRT1 [23, 24]. Therefore, it was hypothesized that USP7 might modulate chondrocytes under inflammation through BiP-eIF2*α*-ATF4-CHOP signaling.

To confirm this hypothesis, the expression of USP7 in the knee articular cartilage of OA mice, caused by the anterior cruciate ligament transection (ACLT), was measured. Functions of USP7 on chondrocyte proliferation, apoptosis, and inflammatory response both in vitro and in vivo were tested, and the underlying mechanisms were explored.

## 2. Materials and Methods

### 2.1. Construction of an OA Mouse Model

The animal study was approved by the Institutional Animal Care and Use Committee of Huazhong Agricultural University (HZAUMO-2020-0014). Male C57BL/6 mice were housed in specific pathogen-free facilities and randomly divided into sham and OA groups. Knee OA models were constructed by ACLT as previously described [25]. Briefly, mice were anesthetized with pentobarbital sodium (70 mg/kg) by intraperitoneal injection. Mice in the OA group received a parapatellar skin incision at the medial side of the right knee joint, the dislocation of the patella, and the ACLT. Mice in the sham group received the joint incision without ACLT.

### 2.2. Haematoxylin-Eosin (HE) Staining, Safranin O-Fast Green Staining, and Immunohistochemical Analysis

Eight weeks later, the knee cartilage tissues were fixed in 4% paraformaldehyde, decalcified in 10% EDTA for 4 weeks, and then sectioned (5 *μ*m). The sections were stained with HE and Safranin O-Fast Green and incubated with of USP7 (1 : 250, Bethyl, AL, USA). Density was measured by Image-Pro Plus software 6.0.

### 2.3. Cell Culture and Induction

ATDC5 cell line was obtained from the Type Culture Collection of the Chinese Academy of Sciences (Shanghai, China). Cells were firstly cultured in a growth medium and then in a chondrogenic-induced medium after 80-90% confluence as our previous study described [26]. Various concentrations of tumor necrosis factor alpha (TNF-*α*) (Novoprotein, Shanghai, China) were added. HBX41108 was from Toris (MN, USA). 4-Phenylbutyric acid (4-PBA) and QNZ were from MCE (NJ, USA).

### 2.4. Cell Transfection of Lentivirus

pLVX-USP7-nc, pLVX-USP7-sh1, and pLVX-USP7-sh2 plasmids were acquired from Miaoling Bioengineering (Wuhan, China). ATDC5 cells were transfected with the three collected lentiviral supernatants as our previous study described [26].

### 2.5. Alcian Blue Staining and Toluidine Blue Staining

Cells were stained by toluidine blue (Aspenbio, Wuhan, China) and alcian blue 8GX (Biosharp, Hefei, China) according to the manufacturer's instructions and observed under the microscope.

### 2.6. CCK-8 Cell Proliferation Assay

Cell proliferation was determined by CCK-8 staining (Dojindo Laboratories, Shanghai, China) according to the manufacturer's instructions.

### 2.7. Quantitative Real-Time Polymerase Chain Reaction (qRT-PCR)

Total RNA was extracted with TRIzol Reagent (Kangwei, Beijing, China). cDNA was harvested with HiScript® III RT SuperMix for qPCR (+gDNA wiper) (Vazyme, Nanjing, China) and measured with ChamQ SYBR qPCR Master Mix (Vazyme, Nanjing, China) in QuantStudio™ 6 Flex Real-Time PCR System (Applied Biosystems, MA, USA). Primer sequences were present in Table 1. The relative mRNA expression was calculated with the 2^−∆∆Ct^ method by normalizing with glyceraldehyde 3-phosphate dehydrogenase (GAPDH).

### 2.8. Western Blot

Cells were lysed with RIPA lysis buffer (Beyotime, Shanghai, China) and phosphate protease inhibitor. Protein samples were harvested, boiled, separated with 12% sodium dodecyl sulphate-polyacrylamide gel electrophoresis, and transferred to polyvinylidene fluoride (PVDF) membranes. The PVDF membranes were blocked with 5% milk and incubated overnight at 4°C with primary antibodies against GAPDH (1 : 2000, ZSGBBIO, Beijing, China), USP7 (1 : 1000, HUABIO, Hangzhou, China), collagen type II alpha 1 chain (Col2a1, 1 : 1000, HUABIO, Hangzhou, China), sex-determining region Y-box 9 (Sox9, 1 : 2000, Abcam, Cambridge, UK), Cleaved Caspase-3 (1 : 1000, CST, MA, USA), Bcl-2 (1 : 1000, ABclonal, Wuhan, China), Bcl-2-associated X (Bax, 1 : 1000, ABclonal, Wuhan, China), eIF2*α* (1 : 1000, ABclonal, Wuhan, China), eIF2*α* phosphorylation (p-eIF2*α*, 1 : 1000, ABclonal, Wuhan, China), activating transcription factor 4 (ATF4, 1 : 1000, HUABIO, Hangzhou, China), CHOP (1 : 300, Santa Cruz, CA, USA), p65 (1 : 1000, CST, MA, USA), p65 phosphorylation (p-p65, 1 : 1000, CST, MA, USA), and proliferating cell nuclear antigen (PCNA, 1 : 1000, HUABIO, Hangzhou, China). Then, they were incubated in horseradish peroxidase-conjugated secondary antibodies (1 : 10000, Biosharp, Hefei, China) and visualized with an electrochemiluminescence reagent.

### 2.9. Immunofluorescence Staining

Cells were harvested and incubated at 4°C overnight with anti-p65 antibodies (1 : 400, CST, MA, USA). Samples were incubated with secondary antibodies conjugated to FITC (ThermoFisher, MA, USA) and DAPI (Beyotime, Shanghai, China) and observed with a fluorescence microscope.

### 2.10. Flow Cytometry

Cell apoptosis was identified with Annexin V-FITC/PI or Annexin V-APC/PI apoptosis detection kits (KeyGEN BioTECH, Nanjing, China) according to the manufacturer's instructions and measured by a flow cytometry (Beckman Coulter, USA).

### 2.11. Transfection of Small Interfering RNA (siRNA)

Cells were transfected with si-CHOP (forward primer 5′-AGCGGAAAGUGGCACAGCUTT-3′, reverse primer 5′-AGCUGUGCCACUUUCCGCUTT-3′) (GenePharma, Suzhou, China) using GP-transfect-Mate (GenePharma, Suzhou, China) and then cultured in the chondrogenic-induced medium and 20 ng/mL TNF-*α* for 48 hours.

### 2.12. Caspase-3 Activity

Caspase-3 activity was detected using the Caspase-3 colorimetric assay kit (KeyGEN BioTECH, Nanjing, China) according to the manufacturer's instructions.

### 2.13. Enzyme-Linked Immunosorbent Assay (ELISA)

IL-6 in the ATDC5 cell supernatant was quantitated by the mouse IL-6 ELISA kit (Neobioscience, Shenzhen, China) according to the manufacturer's instructions.

### 2.14. TUNEL Staining

Cell apoptosis was measured using a TUNEL staining kit (Beyotime, Shanghai, China) according to the manufacturer's instructions and observed with a fluorescent microscope.

### 2.15. Statistical Analysis

All experiments were repeated three times independently. The data were presented as means ± standard deviations (SDs). Data were analyzed by Student's *t*-tests or one-way ANOVAs. *p* < 0.05 was considered statistically significant.

## 3. Results

### 3.1. Decreased Expression of USP7 in OA Mice after ACLT

**Figure 1:**
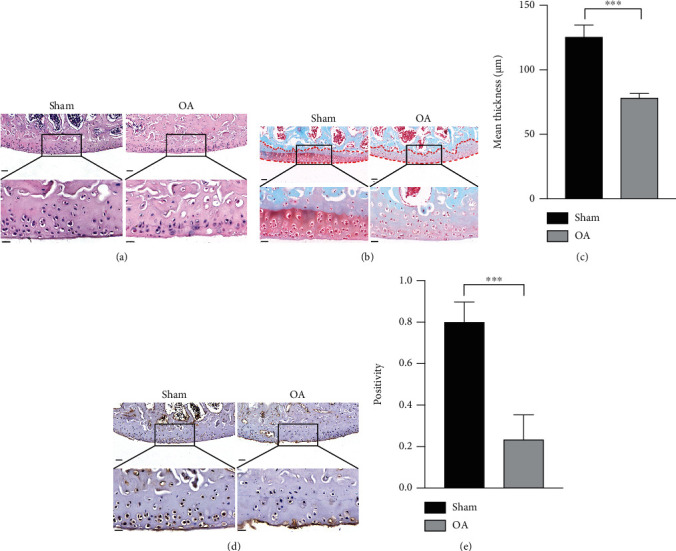
Decreased expression of USP7 in OA mice after ACLT. (a) HE staining of the mouse knee joint after ACLT in the sham and OA groups. (b) Safranin O-Fast Green staining of the mouse knee joint after ACLT in the sham and OA groups. The red dotted line indicates the cartilaginous region of the tibia. (c) Quantitative measurement of (b). (d) Immunohistochemistry staining of the mouse knee joint after ACLT in the sham and OA groups. (e) Quantitative measurement of (d). Scale bars of the upper panels = 50 *μ*m and scale bars of the lower panels = 20 *μ*m. ^∗^*p* < 0.05, ^∗∗^*p* < 0.01, ^∗∗∗^*p* < 0.001, and ^∗∗∗∗^*p* < 0.0001.

Eight weeks after ACLT, all mice in both groups were alive, although those in the OA group showed symptoms of claudication. The HE staining showed that mice in the OA group had fewer chondrocytes, while the sham group had normal morphology (Figure 1(a)). Safranin O-Fast Green staining also showed decreased cartilage thickness and chondrocytes in the OA group (Figures 1(b) and 1(c)), suggesting that OA mouse models were successfully constructed.

Immunohistochemical staining showed that USP7 was mainly situated in the nuclei of the mouse knee joint chondrocytes, and USP7 was decreased in the OA group (Figures 1(d) and 1(e)).

### 3.2. Decreasing ATDC5 Cell Proliferation and Increasing Apoptosis and Inflammatory Response with Increasing TNF-*α*

To mimic OA in vitro, different concentrations of TNF-*α* were added into a chondrogenic-induced medium. Alcian blue and toluidine blue stainings showed that the staining intensities decreased with increasing TNF-*α* (Fig. S1A). ATDC5 cell proliferation was gradually slower with increasing TNF-*α*; however, there was no difference between 20 ng/mL and 40 ng/mL TNF-*α* by CCK-8 assay (Fig. S1B). Cartilage-specific markers, *Col2a1* and *Sox9* mRNA, also decreased with increasing TNF-*α* (Fig. S1C). Western blot revealed that increasing TNF-*α* downregulated Col2a1 protein but upregulated Cleaved Caspase-3 protein (Figs. S1D and E). Caspase-3 activity and the cell apoptosis rate were also upregulated with increasing TNF-*α*, with no difference between 20 ng/mL and 40 ng/mL TNF-*α* (Figs. S1F–H). Proinflammatory cytokines, *IL-6*, *COX*, *NOS2*, and *MMP13* mRNA, had higher expression with increasing TNF-*α* (Fig. S1I). ELISA of IL-6 revealed the same result (Fig. S1J). Considering that 20 ng/mL and 40 ng/mL TNF-*α* showed no obvious differences on chondrocyte proliferation and apoptosis, 20 ng/mL TNF-*α* was selected to mimic OA in the following in vitro experiments.

### 3.3. USP7 Knockdown Inhibits ATDC5 Cell Proliferation and Enhances Apoptosis and Inflammatory Response under TNF-*α*-Induced Inflammation

**Figure 2:**
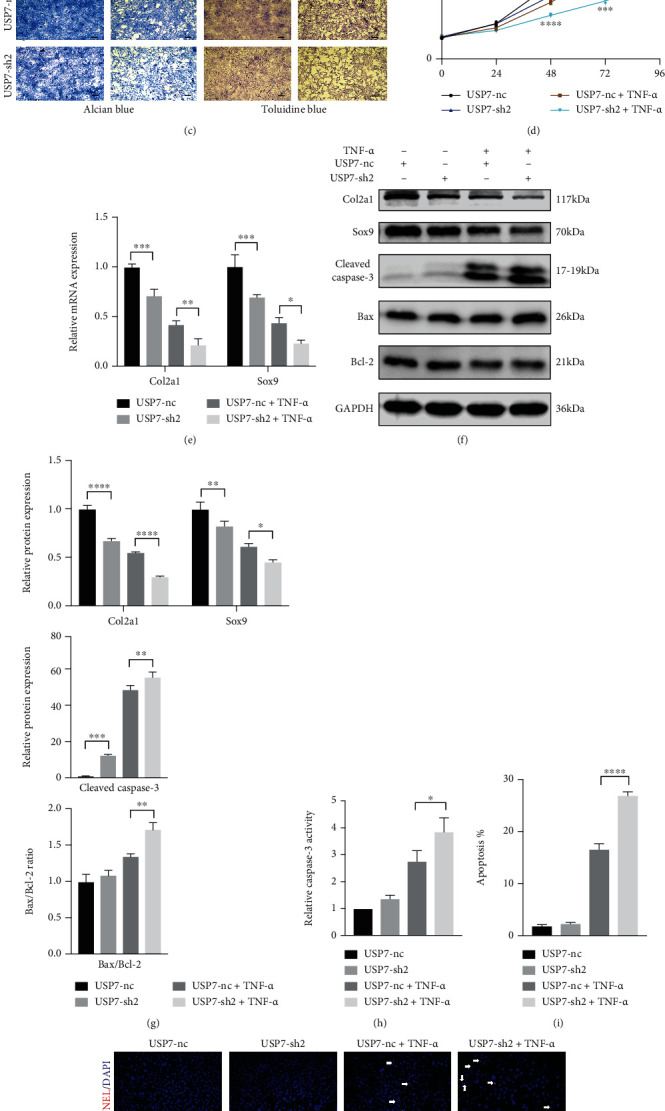
USP7 knockdown inhibits ATDC5 cell proliferation and increases apoptosis and inflammatory response after 48 h chondrogenic induction under TNF-*α*-induced inflammation. (a) Relative *USP7* mRNA expression under 20 ng/mL TNF-*α*. (b) Relative USP7 protein expression in USP7 knockdown and its control groups. (c) Alcian blue and toluidine blue staining in USP7 knockdown and its control groups under TNF-*α*-induced inflammation after 48 h chondrogenic induction. Scale bars = 100 *μ*m. (d) Growth curves in USP7 knockdown and its control groups under TNF-*α*-induced inflammation after 48 h chondrogenic induction measured by CCK-8 assay. (e) Relative *Col2a1* and *Sox9* mRNA expression of in USP7 knockdown and its control groups under TNF-*α* stimulation after 48 h chondrogenic induction. (f) Col2a1, Sox9, Cleaved Caspase-3, Bax, and Bcl-2 protein expression of in USP7 knockdown and its control groups under TNF-*α*-induced inflammation after 48 h chondrogenic induction. (g) Quantitative measurement of (f). (h) Relative Caspase-3 activity in USP7 knockdown and its control groups under TNF-*α*-induced inflammation after 48 h chondrogenic induction. (i) Quantitative measurement of cell apoptosis measured by flow cytometry in the USP7-nc and USP7-sh2 groups under TNF-*α*-induced inflammation after 48 h chondrogenic induction. (j) TUNEL staining in USP7 knockdown and its control groups under TNF-*α*-induced inflammation after 48 h chondrogenic induction. White arrows indicated TUNEL-positive cells. Scale bars = 50 *μ*m. (k) Quantitative measurement of (j). (l) Relative *IL-6*, *COX*, *NOS2*, and *MMP13* mRNA expression in USP7 knockdown and its control groups under TNF-*α*-induced inflammation after 48 h chondrogenic induction. (m) IL-6 expression in USP7 knockdown and its control group supernatant under TNF-*α*-induced inflammation after 48 h chondrogenic induction. ^∗^*p* < 0.05, ^∗∗^*p* < 0.01, ^∗∗∗^*p* < 0.001, and ^∗∗∗∗^*p* < 0.0001.

The USP7 mRNA was reduced under 20 ng/mL TNF-*α* after 48 h chondrogenic induction (Figure 2(a)). USP7 was then knocked down by lentiviral transfection. Positive green fluorescent proteins (GFPs) of ATDC5 cells were over 95%, indicating that they were successfully transfected (Fig. S2). Western blot revealed that USP7 protein was successfully knocked down (Figure 2(b)). Since USP7-sh2 had better knockdown efficiency than USP7-sh1, it was used in the following experiments to determine whether USP7 regulates chondrocyte proliferation, apoptosis, and inflammatory response under TNF-*α*-induced inflammation.

Alcian blue and toluidine blue stainings showed that USP7 knockdown reduced the staining intensities under TNF-*α*-induced inflammation (Figure 2(c)). USP7 knockdown delayed ATDC5 cell proliferation under TNF-*α* (Figure 2(d)). Besides, Col2a1 and Sox9 were lower in the USP7-sh2 group under TNF-*α* (Figures 2(e)–2(g)). To observe functions of USP7 on cell apoptosis, the expression of apoptosis-related proteins, cell apoptosis rate, and TUNEL staining were tested. Western blot showed that USP7 knockdown improved the expression of the Cleaved Caspase-3 protein and the ratio of Bax/Bcl-2 under TNF-*α*-induced inflammation (Figures 2(f) and 2(g)). Caspase-3 activity displayed the same result (Figure 2(h)). Moreover, the ATDC5 cell apoptosis rate was higher in the USP7-sh2 group under inflammation (Figure 2(i) and Fig. S3). TUNEL staining also displayed the same trend (Figures 2(j) and 2(k)). These results indicate that USP7 knockdown upregulated ATDC5 cell apoptosis under TNF-*α*-induced inflammation.

USP7 knockdown also increased the expression of *IL-6*, *COX*, *NOS2*, and *MMP13* mRNA (Figure 2(l)). ELISA showed the same trend of IL-6 (Figure 2(m)), revealing that USP7 knockdown exacerbated the inflammatory response of ATDC5 cells under inflammation.

### 3.4. USP7 Inhibitor HBX41108 Inhibits ATDC5 Cell Proliferation and Enhances Apoptosis and Inflammatory Response under TNF-*α*-Induced Inflammation

HBX41108 was a small molecular inhibitor of USP7. The alcian blue and toluidine blue staining intensities decreased gradually with increasing HBX41108 under TNF-*α* (Fig. S4A). HBX41108 also inhibited ATDC5 cell proliferation at equal or greater than 1 *μ*M in a dose-independent manner (Fig. S4B). Flow cytometry suggested that HBX41108 accelerated chondrocyte apoptosis at a dose-independent manner, except 0.5 *μ*M (Figs. S4C and S4D). Considering that 0.5 *μ*M HBX41108 had no obvious effect on chondrocyte proliferation and apoptosis, 1 *μ*M and 2 *μ*M HBX41108 were selected for use in the following experiment.

HBX41108 inhibited the expression of Col2a1 and Sox9 (Figs. S4E–S4G), upregulated ATDC5 cell apoptosis, and improved the expression of proinflammatory cytokines (Figs. S4F-S4J). These results were consistent with USP7 knockdown. Collectively, USP7 inhibitor HBX41108 reduced chondrocyte proliferation and aggravated apoptosis and the inflammatory response under TNF-*α*-induced inflammation.

### 3.5. USP7 Inhibitor HBX41108 Aggravated Cartilage Destruction of OA Mice

**Figure 3:**
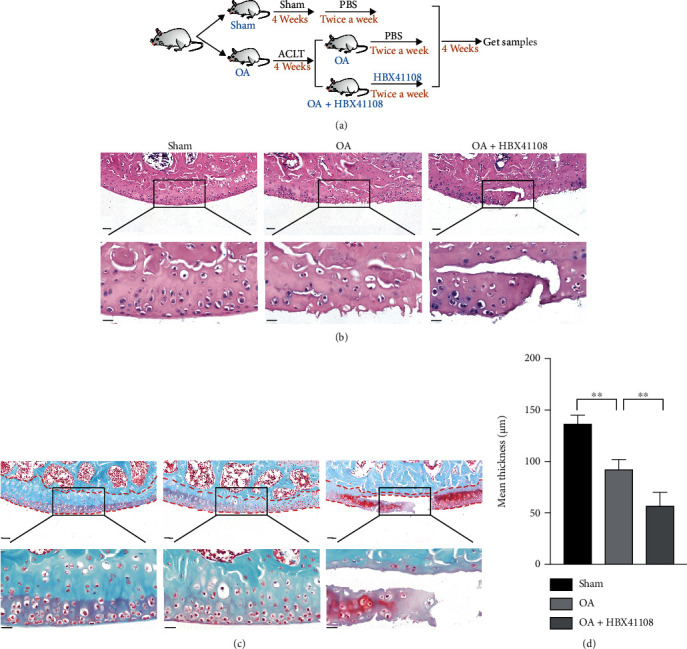
USP7 inhibitor HBX41108 aggravated cartilage destruction of OA mice. (a) Flow chart of the in vivo experiment with HBX41108. (b) HE staining of the mouse knee joint after ACLT in the sham, OA, and OA with HBX41108 groups. (c) Safranin O-Fast Green staining of the mouse knee joint after ACLT in the sham, OA, and OA with HBX41108 groups. The red dotted line indicates cartilaginous region of the tibia. (d) Quantitative measurement of (c). Scale bars of the upper panels = 50 *μ*m and scale bars of the lower panels = 20 *μ*m. ^∗^*p* < 0.05, ^∗∗^*p* < 0.01, ^∗∗∗^*p* < 0.001, and ^∗∗∗∗^*p* < 0.0001.

To determine the functions of USP7 in vivo four weeks after ACLT, the USP7 inhibitor HBX41108 was injected intraperitoneally into the OA mice twice a week for four weeks (Figure 3(a)). The HE staining showed that the OA group with HBX41108 had fewer chondrocytes than the OA group without HBX41108 (Figure 3(b)). Safranin O-Fast Green staining also showed decreased cartilage thickness and structural breakage of cartilage tissue in the OA group with HBX41108 (Figures 3(c) and 3(d)). This indicated that the USP7 inhibitor HBX41108 aggravated cartilage destruction in OA mice.

### 3.6. Knocking Down and Inhibitor HBX41108 of USP7 Activate ERS and NF-*κ*B Signaling under TNF-*α*-Induced Inflammation

**Figure 4:**
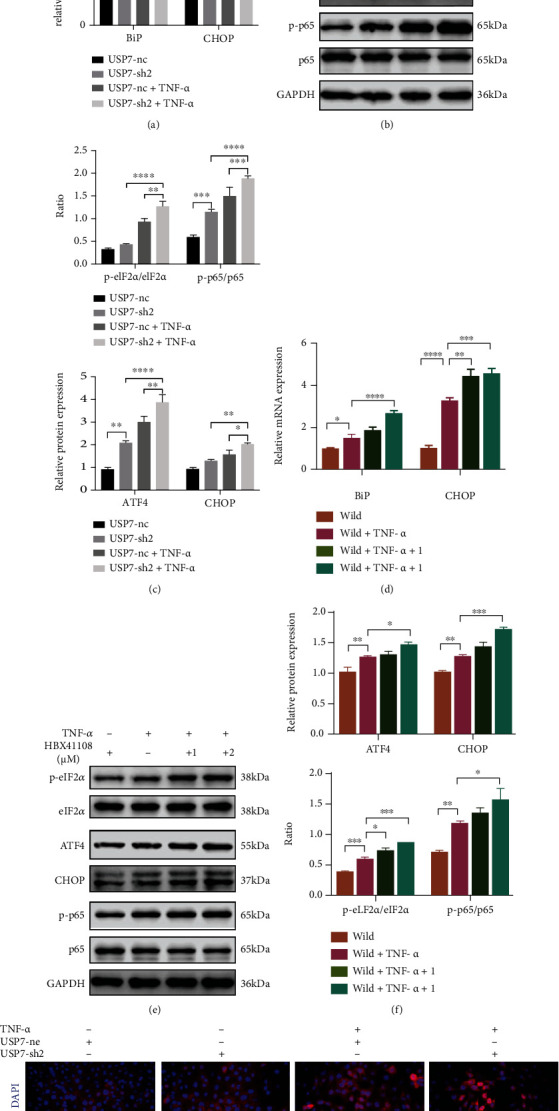
Knocking down and inhibitor HBX41108 of USP7 activate ERS and NF-*κ*B signaling under TNF-*α*-induced inflammation. (a) Relative *BiP* and *CHOP* mRNA expression of in USP7 knockdown and its control groups under TNF-*α*-induced inflammation after 48 h chondrogenic induction. (b) p-eIF2*α*, eIF2*α*, ATF4, CHOP, p-p65, and p65 protein expression of in USP7 knockdown and its control groups under TNF-*α*-induced inflammation after 48 h chondrogenic induction. (c) Quantitative measurement of (b). (d) Relative *BiP* and *CHOP* mRNA expression of wild ATDC5 cells under TNF-*α*-induced inflammation after 48 h chondrogenic induction in HBX41108. (e) p-eIF2*α*, eIF2*α*, ATF4, CHOP, p-p65, and p65 protein expression of wild ATDC5 cells under TNF-*α*-induced inflammation after 48 h chondrogenic induction in HBX41108. (f) Quantitative measurement of (e). (g) Immunofluorescent staining of p65 in USP7 knockdown and its control groups under TNF-*α*-induced inflammation after 48 h chondrogenic induction. Scale bars = 20 *μ*m. ^∗^*p* < 0.05, ^∗∗^*p* < 0.01, ^∗∗∗^*p* < 0.001, and ^∗∗∗∗^*p* < 0.0001.

PCR showed that USP7 knockdown increased*BiP* and *CHOP* mRNA under inflammation (Figure 4(a)). Western blot showed that USP7 knockdown upregulated ATF4 and CHOP proteins, and the ratios of p-eIF2*α*/eIF2*α* and p-p65/p65 under inflammation (Figures 4(b) and 4(c)). Moreover, HBX41108 displayed the same trends (Figures 4(d)–4(f)). Immunofluorescence staining showed that p65 was primarily expressed in the cytoplasm under noninflammation but partially transferred into the nucleus under TNF-*α*-induced inflammation, with more nuclear p65 in the USP7-sh2 group (Figure 4(g)). Collectively, it is suggested that USP7 may through ERS and NF-*κ*B signaling regulate chondrocytes under inflammation. To verify this hypothesis, inhibitors of these two signaling and si-CHOP were used in the following experiments under TNF-*α*-induced inflammation.

### 3.7. ERS Inhibitor 4-PBA Reverses Chondrocyte Proliferation, Apoptosis, and Inflammatory Response Caused by USP7 Knockdown under TNF-*α*-Induced Inflammation

**Figure 5:**
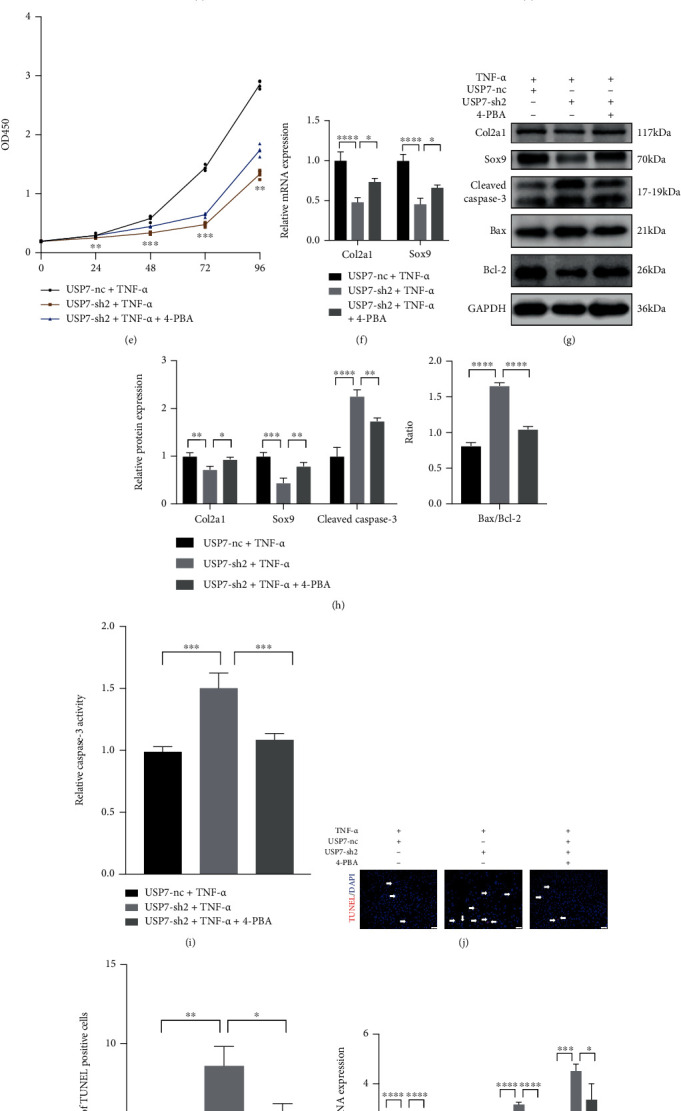
ERS signaling inhibitor 4-PBA reverses chondrocyte proliferation, apoptosis, and inflammation caused by USP7 knockdown under TNF-*α*-induced inflammation. (a) Relative *BiP* and *CHOP* mRNA expression in USP7 knockdown and its control groups under TNF-*α*-induced inflammation after 48 h chondrogenic induction, with and without 4-PBA. (b) p-eIF2*α*, eIF2*α*, ATF4, CHOP, p-p65, and p65 protein expression in USP7 knockdown and its control groups under TNF-*α*-induced inflammation after 48 h chondrogenic induction, with and without 4-PBA. (c) Quantitative measurement of (b). (d) Alcian blue and toluidine blue staining in USP7 knockdown and its control groups under TNF-*α*-induced inflammation after 48 h chondrogenic induction, with and without 4-PBA. Scale bars = 100 *μ*m. (e) Growth curves in USP7 knockdown and its control groups under TNF-*α*-induced inflammation after 48 h chondrogenic induction, with and without 4-PBA, measured by CCK8-assay. (f) Relative *Col2a1* and *Sox9* mRNA expression in USP7 knockdown and its control groups under TNF-*α*-induced inflammation after 48 h chondrogenic induction, with and without 4-PBA. (g) Col2a1, Sox9, Cleaved Caspase-3, Bax, and Bcl-2 protein expression in USP7 knockdown and its control groups under TNF-*α*-induced inflammation after 48 h chondrogenic induction, with and without 4-PBA. (h) Quantitative measurement of (g). (i) Relative Caspase-3 activity in USP7 knockdown and its control groups under TNF-*α*-induced inflammation after 48 h chondrogenic induction, with and without 4-PBA. (j) TUNEL staining in USP7 knockdown and its control groups under TNF-*α*-induced inflammation after 48 h chondrogenic induction, with and without 4-PBA. White arrows indicate TUNEL-positive cells. Scale bars = 50 *μ*m. (k) Quantitative measurement of (j). (l) Relative *IL-6*, *COX*, *NOS2*, and *MMP13* mRNA expression of in USP7 knockdown and its control groups under TNF-*α*-induced inflammation after 48 h chondrogenic induction, with and without 4-PBA. (m) IL-6 expression in USP7 knockdown and its control group supernatant under TNF-*α*-induced inflammation after 48 h chondrogenic induction, with and without 4-PBA. ^∗^*p* < 0.05, ^∗∗^*p* < 0.01, ^∗∗∗^*p* < 0.001, and ^∗∗∗∗^*p* < 0.0001.

4-PBA is a widely used ERS inhibitor. PCR and western blot revealed that 4-PBA effectively inhibited the activated ERS and NF-*κ*B signaling in the USP7-sh2 group under inflammation (Figures 5(a)–5(c)). 4-PBA increased chondrocyte proliferation (Figures 5(d) and 5(e)) and enhanced the expression of Col2a1 and Sox9 (Figures 5(f)–5(h)). Moreover, 4-PBA downregulated chondrocyte apoptosis under inflammation in the USP7-sh2 group (Figures 5(g)–5(k)). 4-PBA also reduced proinflammatory cytokines (Figures 5(l) and 5(m)). These results suggested that 4-PBA reversed chondrocyte proliferation, apoptosis, and inflammatory response caused by USP7 knockdown under TNF-*α*-induced inflammation.

### 3.8. si-CHOP Reverses Chondrocyte Proliferation, Apoptosis, and Inflammatory Response Caused by USP7 Knockdown under TNF-*α*-Induced Inflammation

**Figure 6:**
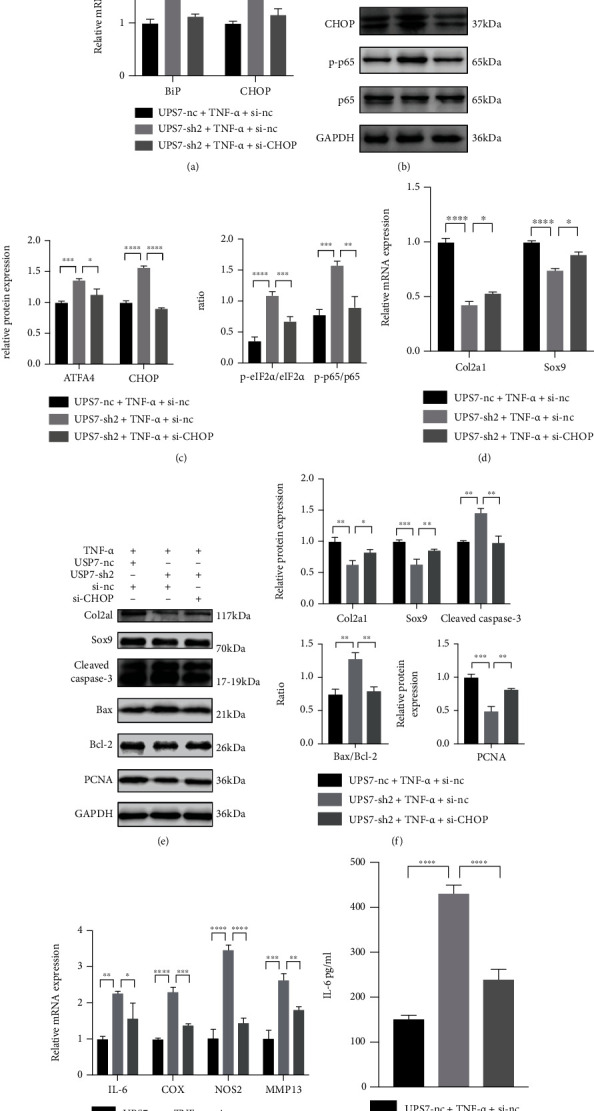
si-CHOP reverses chondrocyte proliferation, apoptosis, and inflammatory response caused by USP7 knockdown under TNF-*α*-induced inflammation. (a) Relative *BiP* and *CHOP* mRNA expression in USP7 knockdown and its control groups under TNF-*α*-induced inflammation after 48 h chondrogenic induction, with and without si-CHOP. (b) p-eIF2*α*, eIF2*α*, ATF4, CHOP, p-p65, and p65 protein expression of in USP7 knockdown and its control groups under TNF-*α*-induced inflammation after 48 h chondrogenic induction, with and without si-CHOP. (c) Quantitative measurement of (b). (d) Relative Col2a1 and Sox9 mRNA expression in USP7 knockdown and its control groups under TNF-*α*-induced inflammation after 48 h chondrogenic induction, with and without si-CHOP. (e) Col2a1, Sox9, Cleaved Caspase-3, Bax, Bcl-2, and PCNA protein expression in USP7 knockdown and its control groups under TNF-*α*-induced inflammation after 48 h chondrogenic induction, with and without si-CHOP. (f) Quantitative measurement of (e). (g) Relative *IL-6*, *COX*, *NOS2*, and *MMP1*3 mRNA expression of in USP7 knockdown and its control groups under TNF-*α*-induced inflammation after 48 h chondrogenic induction, with and without si-CHOP. (h) IL-6 expression in USP7 knockdown and its control group supernatant under TNF-*α*-induced inflammation after 48 h chondrogenic induction, with and without si-CHOP. ^∗^*p* < 0.05, ^∗∗^*p* < 0.01, ^∗∗∗^*p* < 0.001, and ^∗∗∗∗^*p* < 0.0001.

PCR and western blot revealed that si-CHOP efficiently decreased the expression of CHOP (Figures (6)–6(c)). si-CHOP inhibited the activated NF-*κ*B/p65 signaling of USP7 knockdown under inflammation (Figures 6(b) and 6(c)). si-CHOP also upregulated the low expression of Col2a1, Sox9, and PCNA in the USP7-sh2 group (Figures 6(d)–6(f)). Moreover, si-CHOP inhibited the increase of chondrocyte apoptosis and proinflammatory cytokines (Figures 6(e)–6(h)). These indicated that si-CHOP reversed chondrocyte proliferation, apoptosis, and inflammatory response caused by USP7 knockdown under inflammation.

### 3.9. NF-*κ*B Signaling Inhibitor QNZ Reverses Chondrocyte Proliferation, Apoptosis, and Inflammatory Response Caused by USP7 Knockdown under TNF-*α*-Induced Inflammation after Chondrogenic Induction

**Figure 7:**
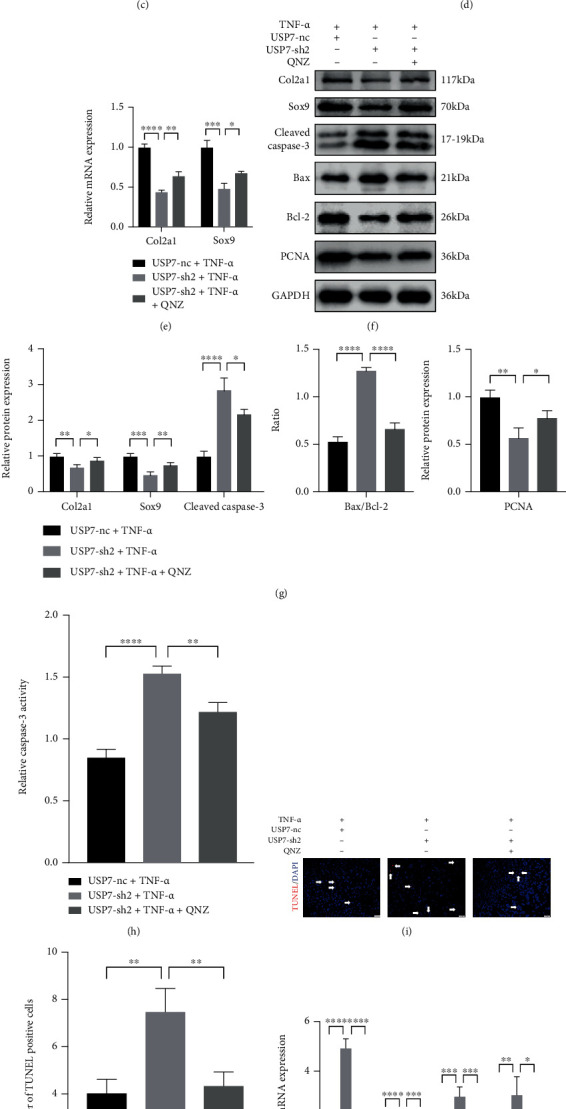
NF-*κ*B signaling inhibitor QNZ reverses chondrocyte proliferation, apoptosis, and inflammatory response caused by USP7 knockdown under TNF-*α*-induced inflammation. (a) Relative *BiP* and *CHOP* mRNA expression in USP7 knockdown and its control groups under TNF-*α*-induced inflammation after 48 h chondrogenic induction, with and without QNZ. (b) p-eIF2*α*, eIF2*α*, ATF4, CHOP, p-p65, and p65 protein expression of in USP7 knockdown and its control groups under TNF-*α*-induced inflammation after 48 h chondrogenic induction, with and without QNZ. (c) Quantitative measurement of B. (d) Alcian blue and toluidine blue staining in USP7 knockdown and its control groups under TNF-*α*-induced inflammation after 48 h chondrogenic induction, with and without QNZ. Scale bars = 100 *μ*m. (e) Relative *Col2a1* and *Sox9* mRNA expression in USP7 knockdown and its control groups under TNF-*α*-induced inflammation after 48 h chondrogenic induction, with and without QNZ. (f) Col2a1, Sox9, Cleaved Caspase-3, Bax, Bcl-2, and PCNA protein expression of in USP7 knockdown and its control groups under TNF-*α*-induced inflammation after 48 h chondrogenic induction, with and without QNZ. (g) Quantitative measurement of F. (h) Relative Caspase-3 activity in USP7 knockdown and its control groups under TNF-*α*-induced inflammation after 48 h chondrogenic induction, with and without QNZ. (i) TUNEL staining in USP7 knockdown and its control groups under TNF-*α*-induced inflammation after 48 h chondrogenic induction, with and without QNZ. White arrows indicated TUNEL-positive cells. Scale bars = 50 *μ*m. (j) Quantitative measurement of (i). (k) Relative *IL-6*, *COX*, *NOS2*, and *MMP1*3 mRNA expression in USP7 knockdown and its control groups under TNF-*α-*induced inflammation after 48 h chondrogenic induction, with and without QNZ. (l) IL-6 expression in USP7 knockdown and its control group supernatant under TNF-*α*-induced inflammation after 48 h chondrogenic induction, with and without QNZ. ^∗^*p* < 0.05, ^∗∗^*p* < 0.01, ^∗∗∗^*p* < 0.001, and ^∗∗∗∗^*p* < 0.0001.

The QNZ was a strong inhibitor of NF-*κ*B signaling. QNZ effectively inhibited the activated ERS and NF-*κ*B signaling of USP7 knockdown under TNF-*α*-induced inflammation (Figures 7(a)–7(c)). Histological stainings showed that QNZ reversed the reduced chondrocytes and extracellular cartilage matrix formation of USP7 knockdown (Figure 7(d)). The expression of Col2a1, Sox9, and PCNA showed the same trend (Figures 7(e)–7(g)). Further, QNZ downregulated the increased chondrocyte apoptosis and proinflammatory cytokines of USP7 knockdown under inflammation (Figures 7(f)–7(l)). These results implied that QNZ reversed chondrocyte proliferation, apoptosis, and inflammatory response of USP7 knockdown under TNF-*α*-induced inflammation.

## 4. Discussion

Posttraumatic OA is caused by joint trauma like anterior cruciate ligament (ACL) injury and meniscus tears [27]. Acute ACL injury caused serious local inflammation in the joint, inducing the proteolysis of aggrecan and Col2a1. Although joint trauma leads to OA in only 12% of cases, it mainly troubles young patients and causes long-term implications [28]. Hence, research on reducing cartilage destruction after ACT injury for OA patients has significant implications.

Increasing evidence suggests that deubiquitinases are essential in bone metabolism; however, their roles in cartilage and related diseases are poorly understood. Our previous study demonstrated that USP7 stimulated chondrocyte proliferation and chondrogenic differentiation in vitro and in vivo [26]. In this study, the knee OA model of mice was successfully constructed by ACLT, and USP7 was found to reduce in the knee joint of OA mice. Continuous local TNF-*α* levels were observed in the injured joint and provided continued stimulation to the surrounding cells [29]. To further study the influence of USP7 on chondrocytes under inflammation and the underlying mechanisms, TNF-*α* was supplemented to mimic OA in vitro. The results revealed that under TNF-*α*-induced inflammation in vitro, inactivation of USP7 inhibited chondrocyte proliferation and accelerated chondrocyte apoptosis and inflammatory response through activation of ERS and NF-*κ*B signaling.

Recent studies confirm that USP7 and its inhibitors regulate cell proliferation and apoptosis, especially in cancers. Inhibition of USP7 reduced cell growth of esophageal squamous cell carcinoma and activated ERS to induce NOXA-mediated apoptosis [30]. In this study, USP7 knockdown activated chondrocyte apoptosis and suppressed chondrocyte proliferation under TNF-*α*-induced inflammation. Further, the USP7 inhibitor HBX41108 showed the same trend in vitro. However, this result differs from a study that found USP7 inhibition alleviated hydrogen peroxide-induced injury, like reduced proliferation and increased inflammation, by regulating NOX4 ubiquitination in rat chondrocytes [31]. Supplementation of hydrogen peroxide or TNF-*α* can mimic OA in vitro, but it led to different expressions and functions of USP7, possibly because USP7, as a deubiquitinase, modulates deubiquitination of diverse proteins to exert its functions under various stimulations. Moreover, the simulated conditions in vitro are not the same as those in vivo, given that the USP7 inhibitor HBX41108 was injected intraperitoneally in vivo and resulted in HBX41108 exacerbated cartilage destruction in OA mice. Hence, these results strongly indicate that USP7 protects cartilage and reduces its destruction in posttraumatic OA.

ERS is regarded as necessary in cartilage development. Chondrocytes, the only cell type in cartilage, showed impaired growth and upregulated apoptosis under ERS-induced conditions [32]. In human OA, ERS contributed to enhanced chondrocyte apoptosis and increased CHOP expression in chondrocytes [33]. Our previous study also found that CHOP suppressed ATDC5 cell proliferation and differentiation after chondrogenic induction [34]. However, the current study found that USP7 promoted ATDC5 cell proliferation. Considering that CHOP is the later event of BiP-PERK-eIF2*α*-ATF4 axis of ERS [35], it is suggested that USP7 may exert its effects through inhibiting BiP-eIF2*α*-ATF4-CHOP signaling.

The results of this study confirmed the supposition. Firstly, it was found that USP7 knockdown and its inhibitor activated BiP-eIF2*α*-ATF4-CHOP signaling of ERS. And then, reduction of ERS with its inhibitor, 4-PBA, could partially rescue the inhibition of chondrocyte proliferation, the promotion of chondrocyte apoptosis, and inflammatory response caused by USP7 knockdown. A recent study also found that administration of 4-PBA alleviated ERS, decreasing the chondrocyte apoptosis in mouse knee joints with obesity-linked OA [36]. According to these results, 4-PBA may be used to treat human OA, but this needs to be investigated further. Furthermore, the current study found that si-CHOP had the same effect as 4-PBA. This was consistent with the finding of Uehara et al. that CHOP knockout restrained the chondrocyte apoptosis and cartilage destruction in mice with OA [37]. Such results might be explained by that CHOP directly decreased antiapoptotic Bcl-2 protein but increased proapoptotic Bax protein to induce cell apoptosis [38]. However, since there were three ERS signalings and our study only demonstrated that CHOP knockdown decreased the BiP-eIF2*α*-ATF4-CHOP signaling, a study of relationship between CHOP knockdown and other ERS signalings may provide better understanding of ERS in chondrocytes under inflammation. Collectively, USP7 modulates chondrocyte proliferation, apoptosis, and inflammatory response via inhibiting BiP-eIF2*α*-ATF4-CHOP signaling of ERS under TNF-*α*-induced inflammation.

On the other hand, inflammation also leads to ERS in the pathogenesis of inflammatory diseases [39, 40]. It was found that ERS could increase proinflammatory cytokines via NF-*κ*B signaling [41]. NF-*κ*B signaling is crucial in OA, and its activation causes cartilage destruction to aggravate the progression of OA [15, 42]. The current study found that USP7 knockdown or its inhibitor activated NF-*κ*B signaling. Moreover, supplementation of this signaling inhibitor QNZ rescued the chondrocyte proliferation, apoptosis, and inflammatory response caused by USP7 knockdown. Other research also reported that QNZ accelerated chondrocyte proliferation and decreased chondrocyte degeneration by promoting glucose uptake [43]. Taken together, this study demonstrates that USP7 suppresses NF-*κ*B/p65 signaling to regulate chondrocyte proliferation, apoptosis, and inflammatory response under TNF-*α*-induced inflammation.

Additional, small molecule inhibitors of USP7 were considered as potential therapies to delay cancer progression by many researchers [3, 44]. However, the results of this study found that the inhibitor of USP7 aggravated cartilage destruction. Hence, clinical doctors should pay more attention to cartilage if the USP7 inhibitor is used for cancers patients with OA. Besides, 4-PBA and QNZ may be potential drugs to slow cartilage destruction of OA and deserve further investigation.

Anyway, this study was based on the function loss of USP7 since the overexpressed lentivirus plasmid was too large to be successfully transfected into ATDC5 cells. The USP7-overexpressed adenovirus vector might be an alternative to be studied in the future. Another limitation was the lack of in vivo studies to verify the underlying mechanisms of USP7 on OA.

## 5. Conclusions

**Figure 8:**
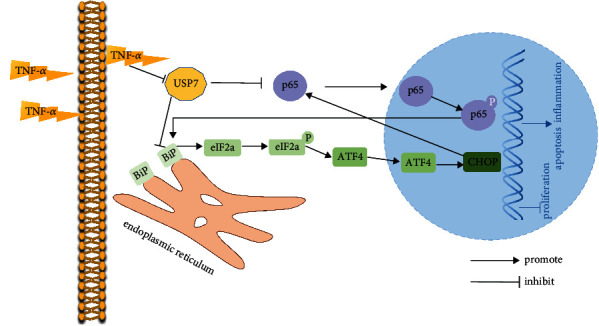
Schematic diagram of the effects and underlying mechanisms of USP7 on chondrocyte proliferation, apoptosis, and inflammatory response under TNF-*α*-induced inflammation. Under TNF-*α*-induced inflammation, USP7 promotes chondrocyte proliferation and suppresses chondrocyte apoptosis and inflammatory response, through inhibiting the BiP-eIF2*α*-ATF4-CHOP signaling of ERS and NF-*κ*B signaling.

In conclusion, this study found that USP7 was reduced at the knee articular cartilage of mice with OA. The knockdown and inhibitor HBX41108 of USP7 hindered chondrocyte proliferation and accelerated chondrocyte apoptosis and inflammatory response under inflammation. USP7 may exert these effects through inhibiting BiP-eIF2*α*-ATF4-CHOP signaling of ERS and NF-*κ*B signaling (Figure 8). This study shows new sights into the effects of USP7 on chondrocyte functions under inflammation.

## Figures and Tables

**Table 1:** Primer sequences for qRT-PCR.

Genes	Forward primer sequence (5′–3′)	Reverse primer sequence (5′–3′)
*GAPDH*	TTGCAGTGGCAAAGTGGAGA	GATGGGCTTCCCGTTGATGA
*USP7*	GCCCTTTGGCCTGTAAATGAG	AGTCTGAGCAACCCCAACAAA
*Sox9*	TGAAGAACGGACAAGCGGAG	CTTGCACGTCGGTTTTGGG
*Col2a1*	CCCGCCTTCCCATTATTGAC	GGGAGGACGGTTGGGTATCA
*IL-6*	AAGACAAAGCCAGAGTCCTTC	TCTGTGACTCCAGCTTATCTGTTA
*COX*	TGCAGAATTGAAAGCCCTCT	CCCCAAAGATAGCATCTGGA
*NOS2*	CTCACTGGGACAGCACAGAA	TGGTCAAACTCTTGGGGTTC
*MMP13*	TGTTTGCAGAGCACTACTTGAA	CAGTCACCTCTAAGCCAAAGAAA
*BiP*	ACTTGGGGACCACCTATTCCT	GTTGCCCTGATCGTTGGCTA
*CHOP*	GCGACAGAGCCAGAATAACAGC	TTCTGCTTTCAGGTGTGGTGGT

## Data Availability

This research article data used to support the finding of this study are included within the article.

## Supplementary Materials

Supplementary Materials Fig. S1: ATDC5 cell proliferation decreases, and apoptosis and inflammation increase under increasing TNF-*α*. (A) Alcian blue and toluidine blue staining of wild ATDC5 cells under increasing TNF-*α* stimulation after 48 h chondrogenic induction. Scale bars = 100 *μ*m. (B) Growth curves of wild ATDC5 cells under increasing TNF-*α* stimulation after 48 h chondrogenic induction. (C) Relative *Col2a1* and *Sox9* mRNA expression in wild ATDC5 cells under increasing TNF-*α* stimulation after 48 h chondrogenic induction. (D) Col2a1 and Cleaved Caspase-3 protein expression in wild ATDC5 cells under increasing TNF-*α* stimulation after 48 h chondrogenic induction. (E) Quantitative measurement of D. (F) Relative Caspase-3 activity in wild ATDC5 cells under increasing TNF-*α* stimulation after 48 h chondrogenic induction. (G) Cell apoptosis measured by flow cytometry in wild ATDC5 cells under increasing TNF-*α* stimulation after 48 h chondrogenic induction. (H) Quantitative measurement of G. (I) Relative mRNA expression of *IL-6*, *COX*, *NOS2*, and *MMP13* in wild ATDC5 cells under increasing TNF-*α* stimulation after 48 h chondrogenic induction. (J) IL-6 expression in wild ATDC5 cell supernatant under increasing TNF-*α* stimulation after 48 h chondrogenic induction. Data were analyzed using one-way ANOVAs. ^∗^*p* < 0.05, ^∗∗^*p* < 0.01, ^∗∗∗^*p* < 0.001, and ^∗∗∗∗^*p* < 0.0001. Fig. S2: ATDC5 cells in USP7 knockdown and its control groups after lentiviral transfection. Scale bars = 100 *μ*m. Fig. S3: cell apoptosis measured by flow cytometry in USP7 knockdown and its control groups under TNF-*α*-induced inflammation after 48 h chondrogenic induction. Fig. S4: USP7 inhibitor inhibits ATDC5 cell proliferation and increases apoptosis and inflammatory response under TNF-*α*-induced inflammation. (A) Alcian blue and toluidine blue staining of wild ATDC5 cells under TNF-*α*-induced inflammation after 48 h chondrogenic induction in USP7 inhibitor HBX41108. Scale bars = 100 *μ*m. (B) Growth curves of wild ATDC5 cells under TNF-*α*-induced inflammation after 48 h chondrogenic induction in HBX41108. (C) Cell apoptosis measured by flow cytometry of wild ATDC5 cells under TNF-*α*-induced inflammation after 48 h chondrogenic induction at different concentrations of HBX41108. (D) Quantitative measurement of C. (E) Relative Col2a1 and Sox9 mRNA expression of wild ATDC5 cells under TNF-*α*-induced inflammation after 48 h chondrogenic induction in HBX41108. (F) Col2a1, Sox9, Cleaved Caspase-3, Bax, and Bcl-2 protein expression of wild ATDC5 cells under TNF-*α*-induced inflammation after 48 h chondrogenic induction in HBX41108. (G) Quantitative measurement of F. (H) Relative Caspase-3 activity of wild ATDC5 cells under TNF-*α*-induced inflammation after 48 h chondrogenic induction in HBX41108. (I) TUNEL staining of wild ATDC5 cells under TNF-*α*-induced inflammation after 48 h chondrogenic induction in HBX41108. White arrows indicated TUNEL-positive cells. Scale bars = 50 *μ*m. (J) Quantitative measurement of I. (K) Relative *IL-6*, *COX*, *NOS2*, and *MMP13* mRNA expression of wild ATDC5 cells under TNF-*α*-induced inflammation after 48 h chondrogenic induction in HBX41108. ^∗^*p* < 0.05, ^∗∗^*p* < 0.01, ^∗∗∗^*p* < 0.001, and ^∗∗∗∗^*p* < 0.0001.
