# Supplementary material for: USP7 Attenuates Endoplasmic Reticulum Stress and NF-κB Signaling to Modulate Chondrocyte Proliferation, Apoptosis, and Inflammatory Response under Inflammation
Source: Oxid Med Cell Longev. 2022 Apr 6;2022:1835900. doi: 10.1155/2022/1835900 (PMC9007692; doi:10.1155/2022/1835900)
Supplement: Supplementary Materials — Fig. S1: ATDC5 cell proliferation decreases, and apoptosis and inflammation increase under increasing TNF-α. (A) Alcian blue and toluidine blue staining of wild ATDC5 cells under increasing TNF-α stimulation after 48 h chondrogenic induction. Scale bars = 100 μm. (B) Growth curves of wild ATDC5 cells under increasing TNF-α stimulation after 48 h chondrogenic induction. (C) Relative Col2a1 and Sox9 mRNA expression in wild ATDC5 cells under increasing TNF-α stimulation after 48 h chondrogenic induction. (D) Col2a1 and Cleaved Caspase-3 protein expression in wild ATDC5 cells under increasing TNF-α stimulation after 48 h chondrogenic induction. (E) Quantitative measurement of D. (F) Relative Caspase-3 activity in wild ATDC5 cells under increasing TNF-α stimulation after 48 h chondrogenic induction. (G) Cell apoptosis measured by flow cytometry in wild ATDC5 cells under increasing TNF-α stimulation after 48 h chondrogenic induction. (H) Quantitative measurement of G. (I) Relative mRNA expression of IL-6, COX, NOS2, and MMP13 in wild ATDC5 cells under increasing TNF-α stimulation after 48 h chondrogenic induction. (J) IL-6 expression in wild ATDC5 cell supernatant under increasing TNF-α stimulation after 48 h chondrogenic induction. Data were analyzed using one-way ANOVAs. ∗p < 0.05, ∗∗p < 0.01, ∗∗∗p < 0.001, and ∗∗∗∗p < 0.0001. Fig. S2: ATDC5 cells in USP7 knockdown and its control groups after lentiviral transfection. Scale bars = 100 μm. Fig. S3: cell apoptosis measured by flow cytometry in USP7 knockdown and its control groups under TNF-α-induced inflammation after 48 h chondrogenic induction. Fig. S4: USP7 inhibitor inhibits ATDC5 cell proliferation and increases apoptosis and inflammatory response under TNF-α-induced inflammation. (A) Alcian blue and toluidine blue staining of wild ATDC5 cells under TNF-α-induced inflammation after 48 h chondrogenic induction in USP7 inhibitor HBX41108. Scale bars = 100 μm. (B) Growth curves of wild ATDC5 cells under TNF-α-induced i [file 1835900.f1.docx]

**Supplementary Figures**


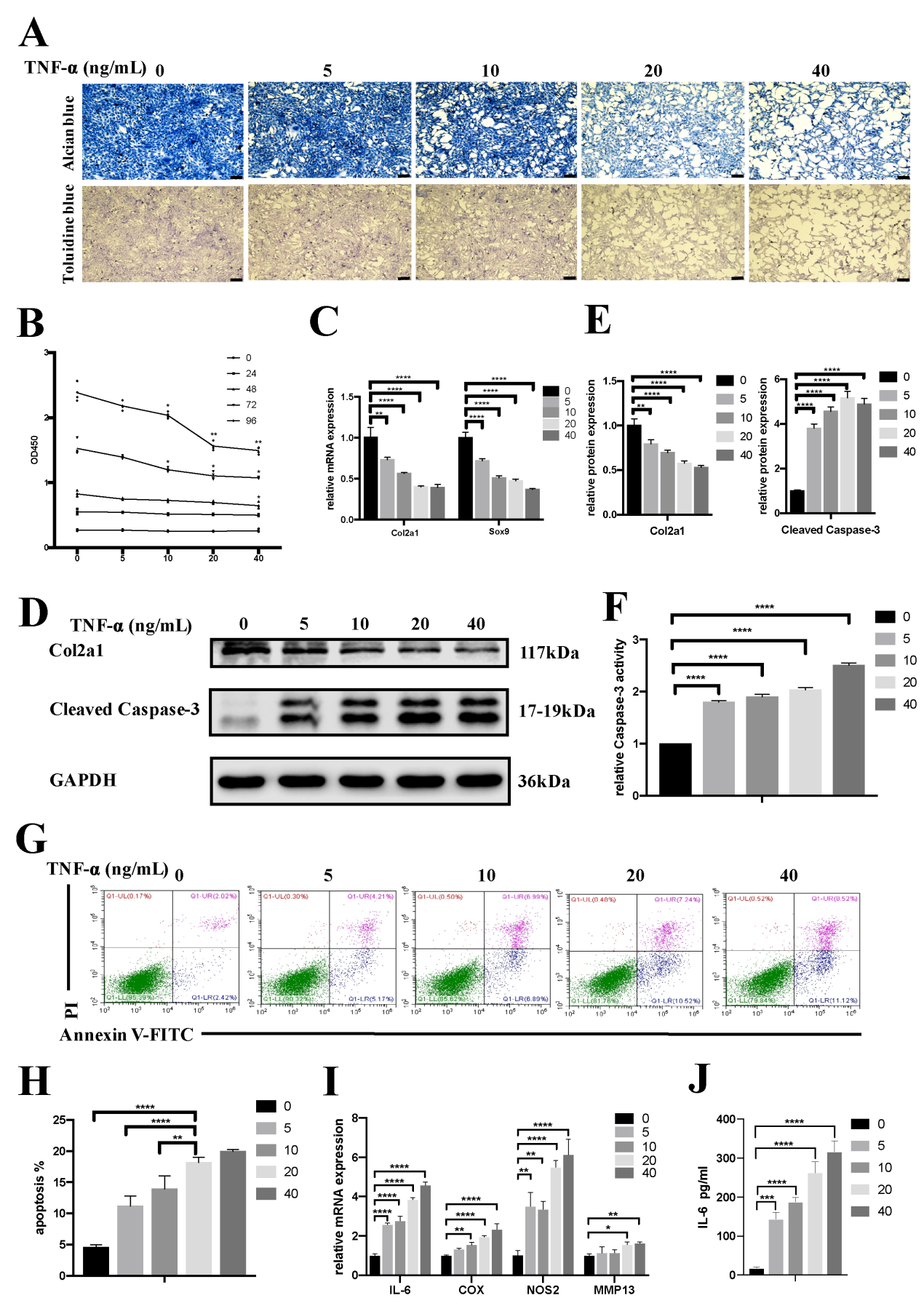


**Fig. S1. ATDC5 cell proliferation decreases, and apoptosis and inflammation increases under increasing TNF-α**


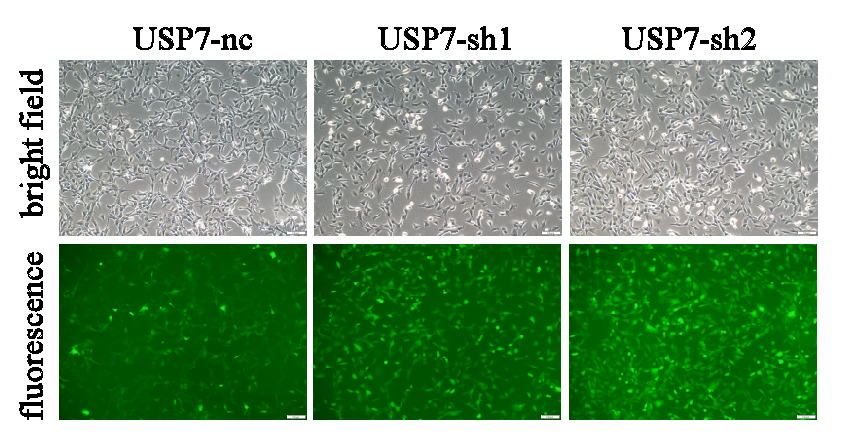


**Fig. S2. ATDC5 cells in USP7 knockdown and its control groups after lentiviral transfection**


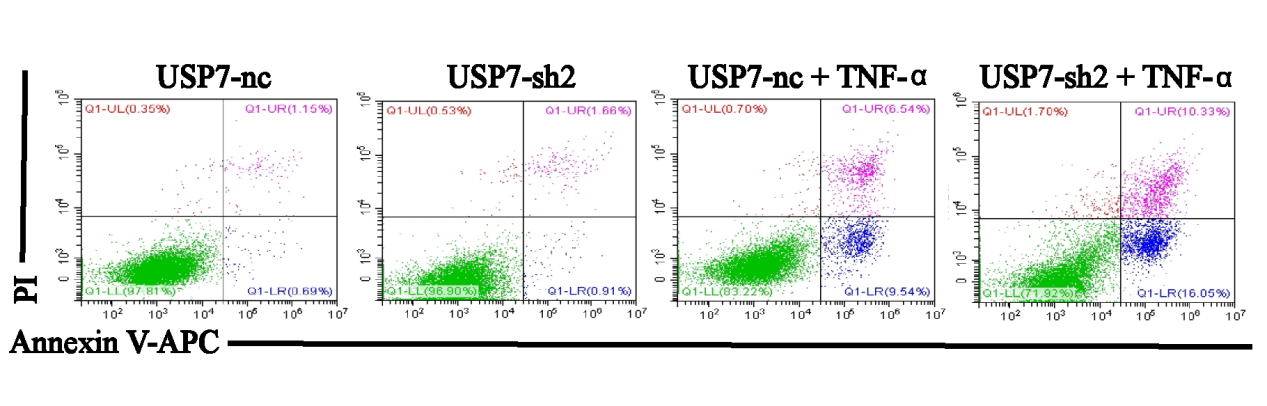


**Fig. S3. Cell apoptosis measured by flow cytometry in USP7 knockdown and its control groups under TNF-α induced inflammation after 48h-chondrogenic induction**


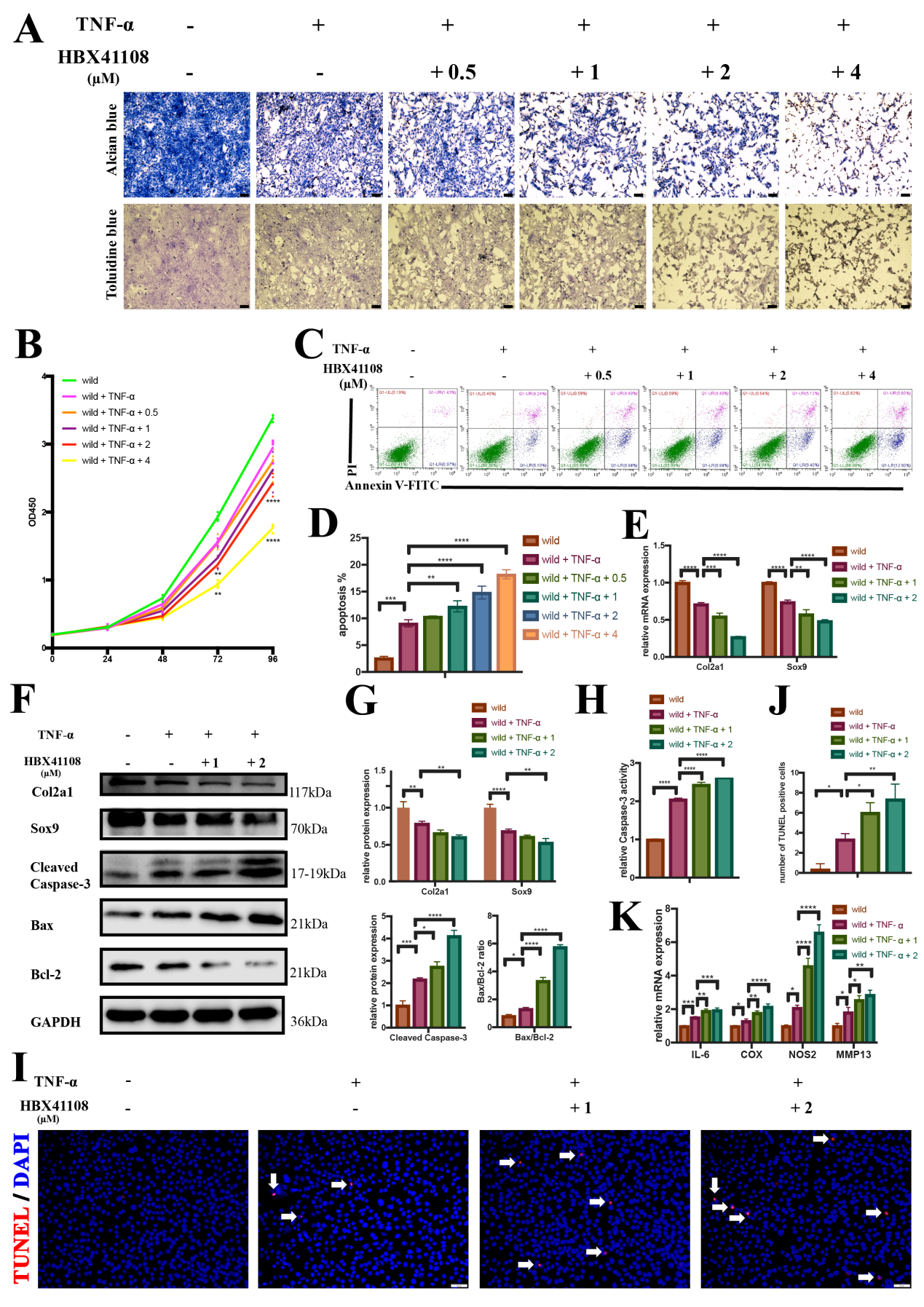


**Fig. S4. USP7 inhibitor inhibits ATDC5 cell proliferation, and increases apoptosis and inflammatory response** **under TNF-α induced inflammation**

**Supplementary Description**

**Fig. S1. ATDC5 cell proliferation decreases, and apoptosis and inflammation increases under increasing TNF-α**

(A) Alcian blue and toluidine blue staining of wild ATDC5 cells under increasing TNF-α stimulation after 48h-chondrogenic induction. Scale bars = 100 μm. (B) Growth curves of wild ATDC5 cells under increasing TNF-α stimulation after 48h-chondrogenic induction. (C) Relative *Col2a1* and *Sox9* mRNAs expression in wild ATDC5 cells under increasing TNF-α stimulation after 48h-chondrogenic induction. (D) Col2a1 and Cleaved Caspase-3 proteins expression in wild ATDC5 cells under increasing TNF-α stimulation after 48h-chondrogenic induction. (E) Quantitative measurement of D. (F) Relative Caspase-3 activity in wild ATDC5 cells under increasing TNF-α stimulation after 48h-chondrogenic induction. (G) Cell apoptosis measured by flow cytometry in wild ATDC5 cells under increasing TNF-α stimulation after 48h-chondrogenic induction. (H) Quantitative measurement of G. (I) Relative mRNA expression of *IL-6*, *COX*, *NOS2*, and *MMP13* in wild ATDC5 cells under increasing TNF-α stimulation after 48h-chondrogenic induction. (J) IL-6 expression in wild ATDC5 cells supernatant under increasing TNF-α stimulation after 48h-chondrogenic induction. Data were analyzed using one-way ANOVAs. **p*<0.05, ***p*<0.01, ****p*<0.001, *****p*<0.0001.

**Fig. S2. ATDC5 cells in USP7 knockdown and its control groups after lentiviral transfection**

Scale bars = 100 μm.

**Fig. S3. Cell apoptosis measured by flow cytometry in USP7 knockdown and its control groups under TNF-α induced inflammation after 48h-chondrogenic induction**

**Fig. S4. USP7 inhibitor inhibits ATDC5 cell proliferation, and increases apoptosis and inflammatory response under TNF-α induced inflammation**

(A) Alcian blue and toluidine blue staining of wild ATDC5 cells under TNF-α induced inflammation after 48h-chondrogenic induction in USP7 inhibitor HBX41108. Scale bars = 100 μm. (B) Growth curves of wild ATDC5 cells under TNF-α induced inflammation after 48h-chondrogenic induction in HBX41108. (C) Cell apoptosis measured by flow cytometry of wild ATDC5 cells under TNF-α induced inflammation after 48h-chondrogenic induction at different concentrations of HBX41108. (D) Quantitative measurement of C. (E) Relative Col2a1 and Sox9 mRNAs expression of wild ATDC5 cells under TNF-α induced inflammation after 48h-chondrogenic induction in HBX41108. (F) Col2a1, Sox9, Cleaved Caspase-3, Bax, and Bcl-2 proteins expression of of wild ATDC5 cells under TNF-α induced inflammation after 48h-chondrogenic induction in HBX41108. (G) Quantitative measurement of F. (H) Relative Caspase-3 activity of wild ATDC5 cells under TNF-α induced inflammation after 48h-chondrogenic induction in HBX41108. (I) TUNEL staining of wild ATDC5 cells under TNF-α induced inflammation after 48h-chondrogenic induction in HBX41108. White arrows indicated TUNEL positive cells. Scale bars = 50 μm. (J) Quantitative measurement of I. (K) Relative *IL-6*, *COX*, *NOS2*, and *MMP13* mRNAs expression of wild ATDC5 cells under TNF-α induced inflammation after 48h-chondrogenic induction in HBX41108. **p*<0.05, ***p*<0.01, ****p*<0.001, *****p*<0.0001.
